# Practical Hints upon Medical Examination for Life Assurance—II

**Published:** 1908-08-08

**Authors:** William P. S. Branson

**Affiliations:** Junior Medical Officer, Sun Life Office.


### Life Assurance.

### PRACTICAL HINTS UPON MEDICAL EXAMINATION FOR LIFE ASSURANCE—II.

By WILLIAM P. S. BRANSON, M.D., M.R.C.P., Junior Medical Officer, Sun Life Office.

If the medical referee wishes to be as helpful as possible he will in making his report bear constantly in mind that the chief-office adviser, to whom will fall the duty of appraising it, is at a great disadvantage in that he has not seen the proposer; for the impression made by the personal inspection of a candidate may modify entirely the bearing of facts revealed by the physical examination. It is of the highest importance, therefore, that the mention of any abnormality, especially one of dubious significance, should be followed by a few words of explanatory comment designed to indicate the view of the abnormality entertained by the actual examiner.

The proposer should always be requested to undress sufficiently to allow of easy access to all parts of the chest, the interval during which he is thus occupied being devoted to a general estimate of the probabilities of the case based upon the physique, aspect, and manner of the proposer. Such an inspection, together with any specific information given in the proposal, will commonly suffice to suggest to the examiner what are the directions in which his examination should be most particular. For example, a full habit of body, suggesting as it does self-indulgence in some form, will prompt careful examination of the tongue, of the liver, and of the urine; conversely, disproportionate lightness of weight in a young subject will urge the need for attention to the examination of the lungs, and special investigation of doubtful causes of death amongst the immediate relatives, and of antecedent illnesses like "influenza," which, as we have said, may mask a phase of pulmonary tuberculosis.

For life-assurance purposes furring of the tongue suggests alcoholism or a foul condition of the mouth; for, although such a circumstance is not unknown, it is extremely unlikely that a man should present himself for this examination when he is febrile. This, of course, is not to say that there are no other alternatives, for there are many; yet the indication is one for care as regards the use of alcohol. Any ulcer of the tongue is in itself a sufficient reason for recommending postponement. Leucoplakia and lingual warts require a full inquiry as to the duration of their existence and the rate of their progression.

General paralysis of the insane, though a relatively infrequent cause of death, is yet a source of heavy losses owing to its rapid fatality. The state of the pupils, both as regards size and mobility to the stimulus of light, should therefore not be neglected. If the pupils be unequal it is of particular importance that their reaction to light should be investigated, for some people have by nature unequal pupils. In these cases the reaction to light is active and natural. But sluggishness or immobility of unequal pupils is an indication for refusal of the life, or at least of protracted postponement. The same may be said of fixity of the pupils without inequality, even though there be no other evidences of tabes.

Rapidity of the pulse, for life-assurance purposes again, indicates emotion, excessive addiction to alcohol or tobacco, or disease of the heart, least often the last. As between the two first of these it may be said that the tachycardia of emotion tends to subside to some extent as the examination proceeds, while the haste attending the second group is enduring and shows little variation. Slowness of the pulse is natural to some people. On the other hand, it may indicate grave disorganisation of the cardiac muscle. Except in the young, notable bradycardia is an indication for postponement unless it can be shown that the condition is habitual. Thickening of the artery and increase in the tension of the pulse will, of course, direct especial attention to the cardio-vascular and urinary examination.

Although examination of the ears need not form a part of the ordinary examination, it is well to bear in mind that otitis media adds a definite risk to life; moreover it is on occasion so chronic and may intrude itself so little upon the consciousness of its victim that mention of it may be omitted, and this without intent to deceive. If, then, the examiner detect symptoms of deafness the tympanic membranes should be investigated for evidences of active disease. If there be a perforation much will turn upon the presence of foul pus on the one hand or of a clean, dry perforation on the other.

In the case of tall thin subjects with poor chests, capacity for expansion is of more moment than actual size. Moreover, it is in such instances as these that the general impression given by the candidate does so much to guide the inferences of the examiner. There are some such people to look at whom is to think of latent phthisis, while others of similar physique have every appearance of health. By appending to the bald figures of chest measurement a commentary on the general aspect and family history the examiner will not only comfort his colleague at the chief office with the assurance that he is on the look-out for likely dangers; but will on occasion obviate the infliction of extra premiums upon lives which do not really deserve them.

There is no point in life-assurance examination which presents more frequent difficulties than the judgment of dubious heart murmurs. Diastolic murmurs at the apex are for the most part organic, and indicate mitral stenosis. They do not as a rule give rise to much difficulty, the conditon being a bar to life assurance unless the case be very favourable and the term quite brief.

Systolic murmurs of maximum intensity in the apical region occur, on the other hand, in great variety, and are the prime source of difficulty in this department of the examination. Sometimes this murmur is clearly organic. It represents the one type of organic valvular disease which life offices, certain of them at least, are willing to accept at an extra rate of premium provided the case is favourable. What, then, constitutes a favourable case of mitral regurgitation? The proposer should be tolerably young, young enough, that is, to justify the assumption that degenerative processes have nothing to do with the lesion: five years at least should have elapsed since the last attack of rheumatic fever, supposing this to have been the known cause of the valvular mischief. The apex-beat should be but little displaced, showing that the added strain upon the heart has been insignificant. The heart-beats should be regular, and reasonably deliberate, and the occupation of the proposer not such as entails violent or prolonged physical effort, nor such as is likely to expose him to a recurrence of rheumatic fever.

*(To be continued.)*

